# The role of radiotherapy in HER2+ early-stage breast cancer patients after breast-conserving surgery

**DOI:** 10.3389/fonc.2022.903001

**Published:** 2023-01-04

**Authors:** Huanzuo Yang, Mengxue Qiu, Yu Feng, Nan Wen, Jiao Zhou, Xiangquan Qin, Juan Li, Xinran Liu, Xiaodong Wang, Zhenggui Du

**Affiliations:** ^1^ Breast Disease Research Center, West China Hospital, Sichuan University, Chengdu, China; ^2^ Department of Breast Surgery, West China Hospital, Sichuan University, Chengdu, China; ^3^ Department of Breast Surgery, Sichuan Academy of Medical Sciences, Sichuan Province People’s Hospital, Chengdu, China

**Keywords:** HER2+ breast cancer, breast-conserving surgery, radiotherapy, radioresistance, nomogram, SEER program

## Abstract

**Background:**

Due to radioresistance, some HER2+ patients may gain limited benefit from radiotherapy (RT) after breast-conserving surgery (BCS). This study aimed to develop an individualized nomogram to identify early-stage HER2+ patients who could omit RT after BCS.

**Methods:**

The data of HER2+ patients with T0-2N0M0 breast cancer after BCS between 2010 and 2015 were extracted from Surveillance, Epidemiology, and End Results (SEER). Based on the independent prognostic factors determined by the Cox analysis in patients without RT after propensity score matching (PSM), the nomogram and risk stratification model were constructed, and then the prognosis of patients with and without RT was compared in each stratified group.

**Results:**

A total of 10799 early-stage HER2+ patients after BCS were included. Baseline characteristics were similar between groups after PSM. Multivariate Cox analysis indicated that RT could improve overall survival (OS) (HR: 0.45, P<0.001) and breast cancer-specific survival (BCSS) (HR: 0.53, P<0.001). Age, marital status, tumor location, tumor size, and chemotherapy were identified by multivariate Cox analysis in patients without RT and were incorporated into a well-validated nomogram. The risk stratification model based on the nomogram indicated that RT was associated with improved OS (HR 0.40, P< 0.001) and BCSS (HR 0.39, P< 0.001) in the high-risk group but not in the low-risk group [OS: HR 1.04, P = 0.94; BCSS: HR 1.06, P = 0.93].

**Conclusion:**

RT could significantly improve the OS and BCSS of HER2+ early-stage breast cancer patients after BCS on the whole. For high-risk patients, RT is an essential component of cancer therapy. However, the omission of radiotherapy may be considered for low-risk HER2+ early-stage patients. Further validation and improvement of the nomogram by prospective study or randomized controlled trials are warranted.

## 1 Introduction

Whether sparing radiotherapy (RT) following breast-conserving surgery (BCS) and which population could be omitted from RT remain highly controversial issues (1). For early-stage breast cancer patients who undergo BCS, postoperative RT is of critical importance. Relevant randomized clinical trials have demonstrated that it could reduce breast tumor local-regional recurrence (LRR) risk and improve overall survival (OS), for example, long-term results of the PRIME-II trial found a 4.1% local recurrence rate in patients who received RT (*vs*. 1.3% in no RT patients) (2–5). Notwithstanding, not all patients gain equal survival benefits due to RT-associated adverse effects and treatment resistance (6, 7). Although proven to be both effective and well-tolerated, the potential toxic effects associated with RT, including adverse breast appearance, dermatitis, breast pain, cardiopulmonary toxicity, and the risk of secondary malignancy, remain concerns (3, 8, 9). The benefits from RT should be weighed against the adverse effects for some breast cancer patients. Heretofore, the CALGB 9343 trial and the PRIME II trial have demonstrated that omitting RT in low-risk elderly patients with low-grade, hormone receptor-positive tumors can be feasible, and recommendations concerning RT omission in those from the NCCN were published in 2017, 2022 and NICE guidelines were published in 2018 (5, 10–13). However, the majority of available studies were only performed on selected elderly patients with receptor-positive tumors, definitive research based on breast cancer patients with other tumor characteristics is still lacking, and more predictive markers are needed for the clinic (1, 6, 14).

HER2 + subtype breast cancer, including non-luminal (HER2 + and ER and PgR-) and luminal (HER2+ and ER or PgR-, or both) breast cancer, exhibits a higher risk of recurrence and a poor prognosis (15). However, trastuzumab has significantly improved the prognosis of HER2+ patients (16, 17). Recently, Cui Y et al. found that HER2+ subtype tumors were significantly enriched in RT resistance (7). This result is consistent with that of a recent randomized trial showing that HER2+ tumors are most radioresistant among all subtypes and thus may gain a limited survival benefit from RT (18). Earlier studies (19–21) also suggested that low-HER2 expression tumors were more likely to respond to RT, which implied that some HER2+ patients who underwent BCS could only attain limited survival benefits compared with patients with other breast cancer subtypes. However, few studies have explicitly explored the effect of RT after BCS in early-stage HER2+ patients because HER2+ patients were excluded from many related studies.

We thus performed a retrospective study aimed to determine whether HER2+ patients after BCS with primary T0–T2N0M0 breast cancer can benefit from RT based on data from the Surveillance, Epidemiology, and End Results (SEER) program. An individualized nomogram including multiple risk factors was established to predict the 3- and 5-year survival of patients without RT, and a risk stratification model was used to further identify a low-risk HER2+ population in whom RT could safely be omitted.

## 2 Materials and methods

### 2.1 Patient data selection

**Figure 1:**
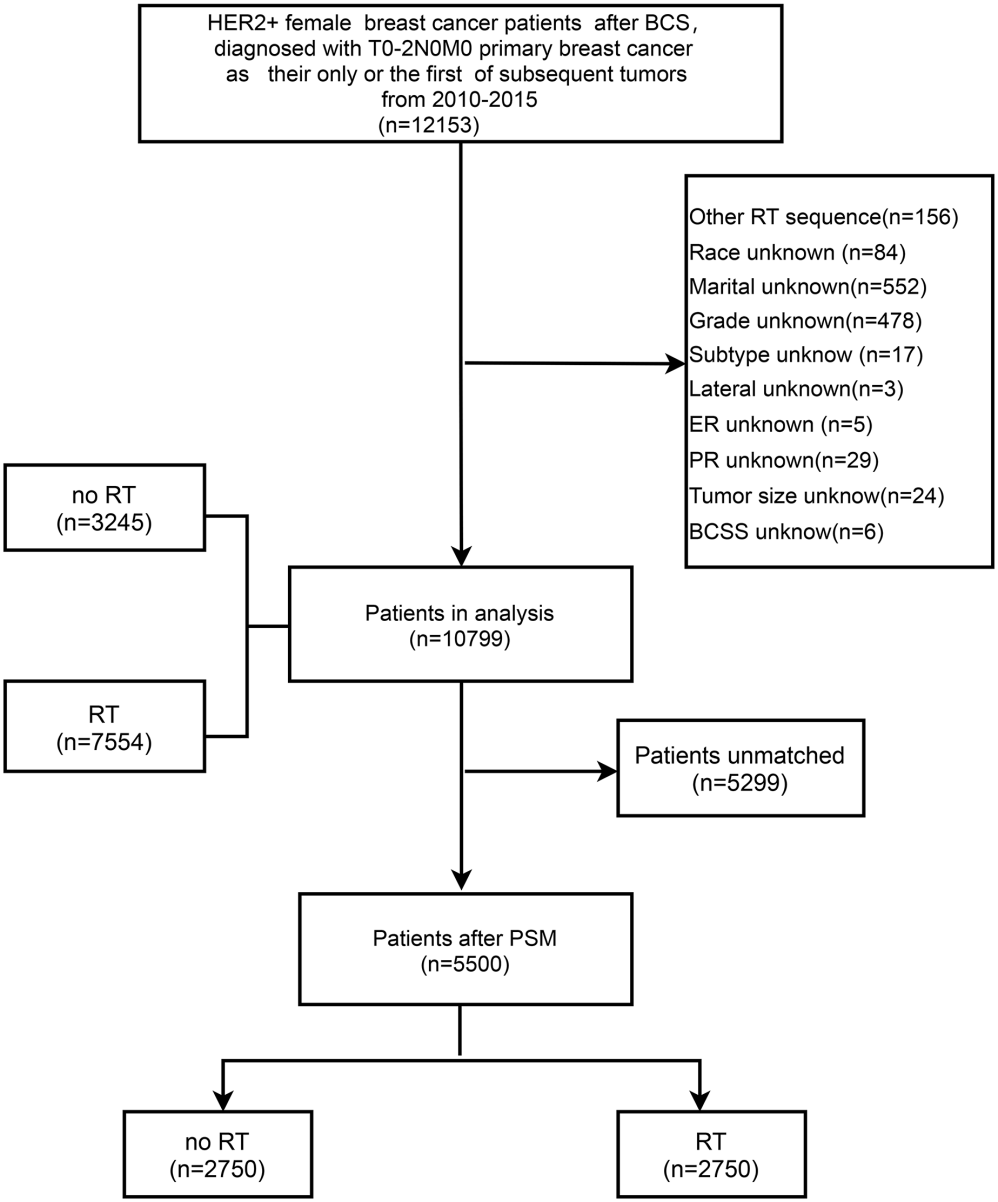
Flow chart of the inclusion and exclusion criteria in the SEER database. BCS, breast-conserving surgery; RT, radiotherapy; PSM, propensity score matching; BCSS, breast cancer-specific survival.

This retrospective study extracted data from the SEER program *via* SEER*Stat software [version 8.3.6 (http://seer.cancer./seer stat)]. Patients were included in this study according to the following criteria: 1) female patients; 2) histological confirmation of breast cancer; 3) HER2+; 4) breast cancer patients after BCS (surgery code in SEER database); 5) T0-T2 N0 M0 (American Joint Committee on Cancer (AJCC) seventh edition TNM staging system; and 6) primary breast cancer as the only or the first subsequent tumor from 2010-2015. The detailed inclusion and exclusion criteria are illustrated in **Figure 1**. We excluded patients preoperatively and intraoperatively treated with RT. Subsequently, we removed patients with unknown or unspecified variable information to reduce information bias.

The variables in this study included demographic characteristics, disease characteristics, treatment characteristics, and survival status, as shown in **Table 1**. Continuous variables, including age at diagnosis and tumor size, were transformed into categorical variables. Using the “number of regional lymph nodes examined” code provided by the SEER program, we used the AJCC definition of a standard axillary lymph node dissection (ALND) (22) as a reference and categorized patients with one to six lymph nodes examined as the sentinel lymph node biopsy (SLNB) group, no node examination or only aspiration of regional nodes as the non-SLNB group, and more than six nodes examined as the ALND group.

**Table 1:** Demographic and disease characteristics of 10799 patients with T0-T2N0M0 HER2+ breast cancer from 2010 to 2015.

Variables	All patients	no Radiotherapy	Radiotherapy	P-value ^c^
	n=10799	(n=3245) n (%)	(n=7554) n (%)	
Age, years				<0.001
<=40	510(4.7)	150(4.6)	360 (4.8)	
40-65	6564(60.8)	1825(56.2)	4739(62.7)	
>=65	3725(34.5)	1270(39.1)	2455(32.5)	
Race				0.143
White	8440(78.2)	2525(77.8)	5915(78.3)	
Blake	1250(11.6)	357(11.0)	895(11.8)	
AIA	58(0.5)	19(0.6)	39(0.5)	
API	1049(9.7)	344(10.6)	705(9.3)	
Marital				<0.001
Unmarried	4195(38.8)	1437(44.3)	2758(36.5)	
Married	6604(61.2)	1808(55.7)	4796(63.5)	
Laterality				0.433
Right	5242(48.5)	1556(48.0)	3686(48.8)	
Left	5557(51.5)	1689(52.0)	3868(51.2)	
Tumor location				0.011
Outer quadrant	4835(44.8)	1381(42.6)	3454(45.7)	
Inner quadrant	2263(21.0)	683 (21.0)	1580 (20.9)	
Center location	398 (3.7)	126 (3.9)	272 (3.6)	
Others ^a^	3303 (30.6)	1055 (32.5)	2248 (29.8)	
Tumor size, cm				<0.001
<=0.5	1350 (12.5)	303 (9.3)	1047 (13.9)	
0.5-1.0	2028 (18.8)	554 (17.1)	1474(19.5)	
1.0-2.0	4361 (40.4)	1387 (42.7)	2974(39.4)	
2.0 +	3060 (28.3)	1001 (30.8)	2059(27.3)	
Histology, ICD-O3				0.029
IDC	9473(87.7)	2804(86.5)	6665(88.2)	
ILC	307(2.8)	96(3.0)	211(2.8)	
IDC+ILC	275(2.5)	82(2.5)	193(2.6)	
Others ^b^	744(6.9)	259(8.0)	485(6.4)	
Grade				0.558
Well; I	826(7.6)	244 (7.5)	582(7.7)	
Moderately, II	4288(39.7)	1267(39.0)	3021(40.0)	
Poorly, III/IV	5685(52.6)	1734(53.4)	3951(52.3)	
Subtype				0.086
HER2+/HR+	8080(74.8)	2392(73.7)	5688(75.3)	
HER2+/HR-	2719(25.2)	853(26.3)	1866(24.7)	
ER				0.073
Negative	2891(26.8)	907(28.0)	1984(26.3)	
Positive	7908(73.2)	2338(72.0)	5570(73.7)	
PR				0.043
Negative	4675(43.3)	1453(44.8)	3222(42.7)	
Positive	6124(56.7)	1792(55.2)	4332(57.3)	
Stage, AJCC 7th				<0.001
IA+0	7742(71.7)	2244(69.2)	5498(72.8)	
IIA	3057(28.3)	1001(30.8)	2056(27.2)	
T category				
Tis+T1mic	316(2.9)	61(1.9)	255(3.4)	
T1	7426(68.8)	2183(67.3)	5243(69.4)	
T2	3057(28.3)	1001(30.8)	2056(27.2)	
Chemotherapy				<0.001
No/unknown	3511(32.5)	1390(42.8)	2121(28.1)	
Yes	7288(67.5)	1855(57.2)	5433(71.9)	
Axillary surgery				<0.001
None	659(6.1)	369(11.4)	290(3.8)	
SLNB	9363(86.7)	2631(81.1)	6732(89.1)	
ALND	777(7.2)	245 (7.6)	532(7.0)	

AIA, American Indian/Alaska Native; API, Asian or Paciﬁc Islander; ILC, invasive lobular carcinoma; HER2, human epidermal growth

factor receptor 2; HR, Hormone receptor; −, negative; +, positive; ER, estrogen receptor; PR, progesterone receptor; IDC, invasive ductal carcinoma; SLNB, sentinel lymph node biopsy; ALND, axillary lymph node dissection.

a “others” includes “tumor location, NOS” and “overlapping lesion of the breast such as 3, 6, 9, 12 o’clock” as recorded in the SEER database.

b “others” means histological types other than the above four types.

c P <0.05 was considered statistically significant.

d Chemotherapy was defined as a risk factor according to a previous study.

### 2.2 Outcome definition

OS referred to the time from the date of diagnosis to death from any cause. We used the cause-specific death classification in the SEER database as BCSS, which referred to the time from the date of diagnosis to the date of death due to breast cancer. Non-BCSS referred to the time from the date of diagnosis to the date of death attributed to causes other than breast cancer based on the other cause of death classification in the SEER database.

### 2.3 Statistical analysis

The correlation between RT and patient characteristics was analyzed using the Pearson chi-square test for categorical variables and the Student t-test for continuous variables. Propensity score matching (PSM) was performed at a 1:1 ratio to adjust baseline characteristics between groups (23). Competing risk analysis was used to evaluate non-BCSS before and after PSM (24). The Kaplan–Meier plots and log-rank tests were applied to determine and compare OS and BCSS.

A nomogram for predicting 3- and 5-year BCSS was constructed based on patients without RT after PSM. Independent prognostic factors were determined by the univariate and multivariate Cox proportional hazards regression models. All variables with P-value <0.1 in univariate Cox analysis were included in multivariate Cox analysis. Variables with a P < 0.05 were included in the final Model. The discrimination of the nomogram was assessed by the concordance index (C-index), and a bootstrap internal validation procedure was performed with 1000 bootstrap resamples to assess the accuracy of the prediction model. Calibration curves were plotted to estimate the consistency between the actual and predicted survival. Based on the nomogram scores, we used the tertiles to determine the optimal cutoff points.

Analyses were performed by Stata/MP version 13.0, SPSS statistical software version 26.0, and R software version 3.6.2. The statistical tests were 2-sided, and P ≤0.05 was considered statistically significant.

## 3 Results

### 3.1 Characteristics of the eligible patients

A total of 10799 eligible patients, including 7554 patients (69.95%) who received RT and 3245 patients (30.05%) who did not receive RT, were included in the study based on the inclusion and exclusion criteria (**Figure 1**). The demographic and clinicopathological characteristics of all involved patients are summarized in **Table 1**. The comparison of patients with and without RT presented substantial differences in age at diagnosis, marital status, tumor location, tumor size, histology, PR status, stage, T category, chemotherapy, and axillary surgery. Regarding the without RT group, patients were mainly distributed in the age band of 40-65, and they tended to have breast cancer in the outer quadrant, relatively small tumor sizes, positive hormone receptor status, and early-stage breast cancer. Compared to the RT group, the frequency of chemotherapy was lower in the non-RT group (71.9% *vs*. 57.2%, P <0.001). After PSM analysis to reduce selection bias, the baseline characteristics were similar between groups (**Supplemental Table 1**), and the distribution of propensity scores for matched and unmatched patients is shown in **Supplemental Figures 1** and **2**.

### 3.2 Analysis of survival benefits from RT

**Figure 2:**
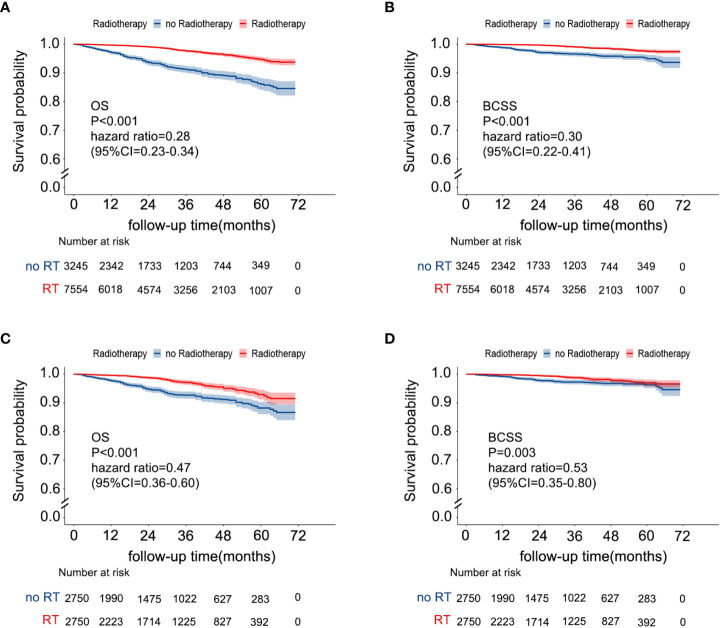
Kaplan–Meier survival curves of the effect of RT on OS [**(A)** before PSM; **(C)** after PSM] and BCSS [**(B)** before PSM, **(D)** after PSM]. RT, radiotherapy; OS, overall survival; BCSS, breast cancer-specific survival; PSM, propensity score matching.

The median follow-up time of 10799 patients was 29 months (IQR, 13-49). During the follow-up period, 408 patients died, 161 of whom died due to breast cancer. Among patients without RT, 233 patients died, of whom only 89 (38.2%) died due to breast cancer. Among patients with RT, 175 patients died, of whom 72 (41.1%) died due to breast cancer. According to the Kaplan-Meier curves (**Figures 2A, B**), patients with RT had better OS (RT *vs*. no RT: unadjusted hazard ratio (HR) 0.28; 95% CI, 0.23–0.34; P< 0.001) and BCSS (RT *vs*. RT: unadjusted HR 0.30; 95% CI, 0.223–0.41; P < 0.001) than patients without RT. In the 5500 patients after PSM, the OS was different in the two groups (no RT *vs* RT: 86.6%, 95% CI 83.9-89.3%;91.5%,95% CI 89.5-93.5, P <0.001). Breast cancer-free survival was 94.6% (95% CI 92.4-96.8) in women allocated to no RT and 96.6% (95.2-98.0) in those assigned to RT. Multivariate Cox analysis indicated that RT was still a protective factor (RT *vs*. no RT: adjusted HR of OS 0.45; 95% CI, 0.35-0.58; P < 0.001; adjusted HR of BCSS 0.530; 95% CI, 0.35–0.80; P=0.002), as shown in **Supplemental Table 2** and **Figures 2C, D**.

### 3.3 Competitive risk analysis of BCSS and non-BCSS

**Figure 3:**
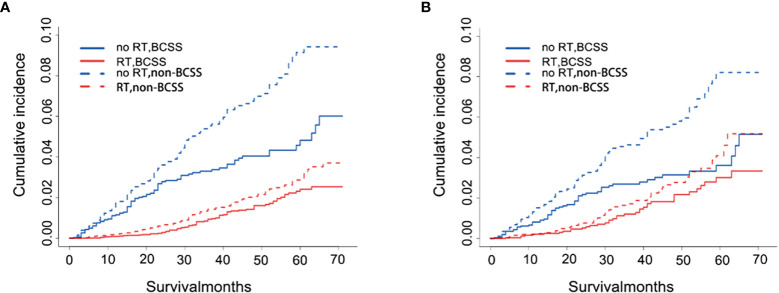
Cumulative incidence plot depicting BCSS and non-BCSS based on RT before **(A)** and after **(B)** PSM. RT, radiotherapy; BCSS, breast cancer-specific survival; non-BCSS, non-breast cancer-specific survival.

These results of competing risk analysis were shown in **Supplemental Tables 3**, **4** and **Figure 3A**. After 71 months of follow-up, the 5-year cumulative incidences of BCSS and non-BCSS in patients were both lower than that in patients without RT [(BCSS:2.4% *vs*. 4.8%; HR,0.30,95%CI,0.22-0.42; P<0.001); (non-BCSS: 2.9% *vs*. 9.1%%; HR,0.27;95%CI,0.27-0.35; P<0.001)]. The result after PSM is shown in **Supplemental Tables 3**, **5** and **Figure 3B**. The difference in the 5-year BCSS rates between the two groups narrowed (no RT *vs*. RT, 3.6% *vs*. 3.0%) after controlling for confounders but was still significant (HR:0.53;95%CI,0.35-0.80, P=0.003). Nevertheless, the 5-year non-BCSS risk still obviously differed (no RT *vs* RT, 8.2% *vs*. 4.1%; HR,0.43;95%CI,0.31-0.60, P<0.001).

### 3.4 Construction of the nomogram and validation in patients without RT

**Figure 4:**
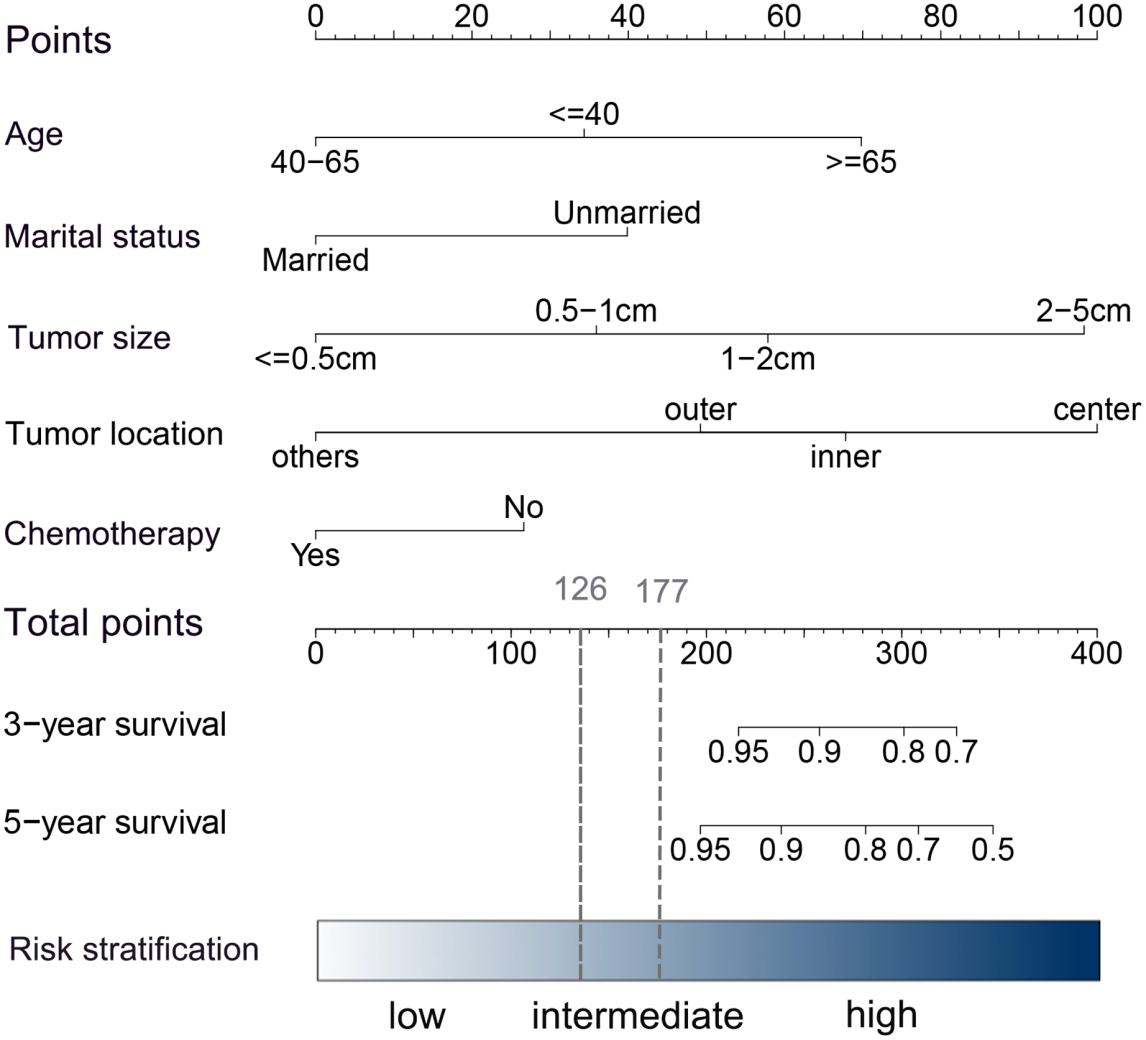
Nomogram for predicting 3- and 5-year BCSS in HER2+ patients with early-stage breast cancer after BCS. In the nomogram, each risk covariate is assigned a score according to the clinicopathological features of an individual patient on the points scale. By summing these scores, the total score can be obtained. Higher scores indicate a higher risk.

Univariate and multivariate Cox proportional hazards regression models were performed to determine the independent risk factors associated with BCSS in patients without RT after PSM. Age at diagnosis, marital status, tumor location, and tumor size were identified as independent prognostic factors related to BCSS in multivariate analysis (P<0.05) (**Table 2**). Chemotherapy, identified as an important prognosis factor (25), was included in the nomogram along with the above 4 factors to predict the 3- and 5-year BCSS of patients without RT (**Figure 4**). According to the point scale in the nomogram, scores were assigned for each variable (**Supplemental Table 6**). The external validation was carried out in patients who received RT after PSM.

**Table 2:** Univariate and multivariate Cox models for BCSS in HER2+ breast cancer patients without RT after PSM (n=2750).

Variables	Univariate ^§^		multivariate	
	HR (95%CI)	P-value ^b^	HR (95%CI)	P-value ^c^
Age, years		<0.001		<0.001
<=40	1.00 [Reference]		1.00 [Reference]	
40-65	0.56(0.13-2.43)	0.44	0.55(0.13-2.41)	0.429
>=65	2.25(0.54-9.31)	0.264	1.85(0.44-7.88)	0.403
Marital				
unmarried	1.000 [Reference]		1.00 [Reference]	
married	0.430(0.26-0.73)	0.002	0.50(0.30-0.85)	0.01
Tumor Location		0.016		0.005
Outer quadrant	1.00 [Reference]		1.00 [Reference]	
Inner quadrant	1.44(0.80-2.60)	0.221	1.38(0.77-2.49)	0.284
Center	2.12 (0.74-6.06)	0.162	2.41(0.84-6.95)	0.103
Others^a^	0.45(0.21-0.96)	0.038	0.43(0.20-0.91)	0.027
Tumor size, cm		0.021		0.004
<=0.5	1.00 [Reference]		1.00 [Reference]	
0.5-1.0	1.83(0.38-8.80)	0.452	1.87 (0.39-9.02)	0.437
1.0-2.0	2.40(0.57-10.16)	0.236	2.73(0.64-11.74)	0.176
2.0+	4.50(1.07-18.91)	0.040	5.51(1.29-23.56)	0.021
Stage, AJCC 7th				
IA+0	1.00[Reference]			
IIA	2.17(1.30-3.62)	0.003		
T category				
Tis+T1	1.00 [Reference]			
T2	2.17(1.30-3.62)	0.003		
Chemotherapy				
No/Unknown	1.00 [Reference]		1.00 [Reference]	0.095^d^
Yes	0.54(0.33-0.91)	0.019	0.63(0.37-1.08)	
Axillary Surgery		0.077		
None	1.00 [Reference]			
SLNB	0.38 (0.16-0.88)	0.024		
ALND	0.40 (0.11-1.42)	0.155		

95% CI, 95% confidence interval; AIA, American Indian/Alaska Native; API, Asian or Paciﬁc Islander; ILC, invasive lobular carcinoma; HER2, human epidermal growth factor receptor 2; HR, Hormone receptor; −, negative; +, positive; ER, estrogen receptor; PR, progesterone receptor; IDC, invasive ductal carcinoma; SLNB, sentinel lymph node biopsy; ALND, axillary lymph node dissection.

^§^ Only P values < 0.1 are listed.

a “others” includes “tumor location, NOS” and “overlapping lesion of the breast such as 3, 6, 9, 12 o’clock” as recorded in the SEER database.

b P <0.01 was considered statistically significant.

c P < 0.05 was considered statistically significant.

d Chemotherapy was defined as a risk factor according to a previous study.

The internal and external validation of the model was carried out by the bootstrap sample validation method and exhibited sufficient accuracy. The C-index of the nomogram was 0.77 (95% CI, 0.71-0.83) in the internal validation and 0.76(95% CI,0.69-0.83) in the external validation, implying that the model had good discriminative ability (**Supplemental Table 7**). The calibration curves for 3- and 5-year BCSS indicated good probability consistencies between the predicted and observed outcomes (**Supplemental Figure 3**).

### 3.5 Survival analysis of risk stratification group

**Figure 5:**
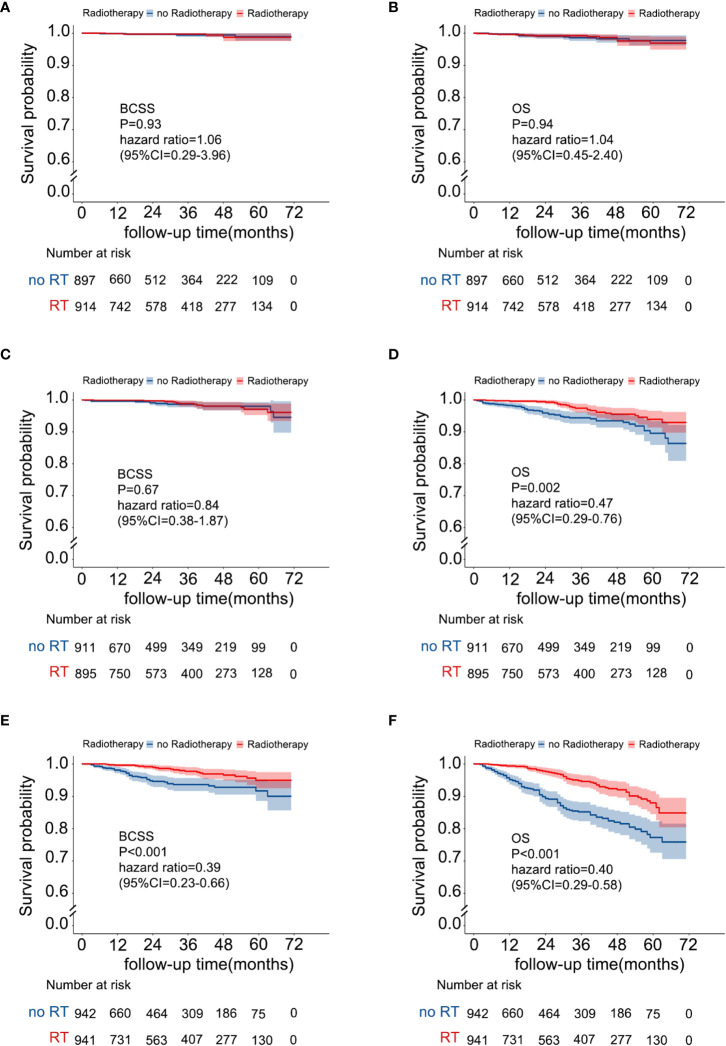
Survival benefit from RT in the risk stratification groups. **(A)** BCSS in the low-risk group, **(B)** OS in the low-risk group, **(C)** BCSS in the intermediate-risk group, **(D)** OS in the intermediate-risk group, **(E)** BCSS in the high-risk group, and **(F)** OS in the high-risk group.

A risk stratification model based on the nomogram (**Figure 4**) for predicting BCSS in patients without RT after PSM was built, and total nomogram scores were calculated. Afterward, the cutoffs of the risk stratification model were defined as 126 and 177, which correspond to the tertiles of the nomogram score in patients without RT after PSM. Following the same scoring method, the total score of patients with RT after PSM was simultaneously calculated. Subsequently, according to the risk stratification model, patients after PSM were stratified into three risk groups: low-risk group (total nomogram score <126; 1811/5500, 32.9%), intermediate-risk group (total nomogram score>=126 and <177; 1806/5500, 32.8%), and high-risk group (total nomogram score>=177; 1883/5500, 34.2%). The Kaplan–Meier plot (**Figures 5A–F**) and log-rank test for the risk stratification model showed that RT was significantly associated with improved OS (HR 0.40; 95% CI, 0.29-0.56; P< 0.001) and BCSS (HR 0.39; 95% CI, 0.23-0.66; P< 0.001) in patients in the high-risk group but not in those in the low-risk group [OS: HR 1.04; 95% CI, 0.45–2.40; P = 0.94; BCSS: HR 1.06; 95% CI, 0.29–3.96; P = 0.93]. In the intermediate-risk group, RT improved BCSS [HR 0.84; 95% CI, 0.38–1.87; P =0.67] but did not improve OS [HR 0.47; 95% CI, 0.29-0.76; P= 0.002].

## 4 Discussion

Considerable studies (2–4, 6, 10) have demonstrated that RT after BCS is a well-established treatment, resulting in a decreased LRR rate and mortality risk. Nevertheless, HER2+ breast cancer is heterogeneous, and not all patients respond equally to RT (7, 18, 19). Much previous evidence supports the role of HER2 in RT resistance, and HER2+/CD44+/CD24−/low cells, Fak activation *in vitro* and *in vivo*, and STAT3-survivin signaling may be associated with RT resistance in HER2+ breast cancer patients (26–28). Therefore, for patients less likely or unlikely to respond to RT, omitting RT would prevent adverse effects from occurring with no therapeutic yield. In the current study, the results of multivariate Cox analysis after PSM indicated that RT could significantly improve the OS and BCSS of HER2+ patients after BCS. However, according to further stratification analysis, we found that RT was beneficial for high-risk patients to improve OS and BCSS, while patients in the remaining groups seemed to derive no survival benefit from RT, especially those in the low-risk group. The side effects of radiotherapy (due to scattered irradiation of nearby vital organs) may wipe out any benefit, which means that it should be possible to define a subgroup for whom RT after BCS can feasibly be omitted.

Although LRR is often used to evaluate the efficacy of RT after BCS, our nomogram mainly focused on BCSS, and OS was also predicted in the stratification model. First, although the SEER database did not provide information about LRR, BCSS was sufficiently reliable and effective to evaluate the treatment effect of RT and could indirectly reflect LRR. Two meta-analyses (2, 4) from the Early Breast Cancer Trialists’ Collaborative Group (EBCTCG) indicated that for patients who received RT, one breast cancer death was avoided by year 15 for every four recurrences avoided by year 10, and the 15-year overall mortality should be reduced. Furthermore, BCSS may more objectively and intuitively reflect the absolute survival benefit of RT. In the competing analysis, we found that the 5-year cumulative incidence of non-BCSS in patients who received RT was lower than those without RT (HR,0.43;95%CI,0.31-0.60, P<0.001). This finding may indicate that OS in patients who received RT was prolonged mainly due to the selection effect of RT, which means that patients who are healthier and who tolerate adverse effects well are more likely to be offered RT than those who are more likely to have comorbid diseases. A similar result was observed by Johnson ME et al, even though they focused on RT after mastectomy (29).

In our risk stratification model constructed with the nomogram, the low-risk group, which was characterized by younger age (40–65), marital status, outer quadrant tumors, and small tumor size, did not benefit from RT. Age is highly correlated with the prognosis of breast cancer. Patients aged <40 years at diagnosis are generally considered to have a higher mortality rate due to more aggressive tumor biology, such as larger tumor size and higher grade (30). In patients aged >= 40 years, in contrast, they are more likely to display lower-risk tumors, but those with a relatively younger age are associated with better somatic function and are more likely to receive adequate treatment, resulting in a better OS and BCSS, compared with elderly patients (31, 32). Similar to the finding of a previous study, married cancer patients had a more favorable prognosis than unmarried patients. The reasons underlying this association are not fully understood, and some researchers believe it may be related to better social, financial, and emotional support and early-stage diagnosis in married cancer patients (33). Additionally, in accordance with the results, we found that patients with tumors in the outer quadrant had a significantly better prognosis than those with tumors in the inner quadrant or center location. This may be because the outer quadrant is a favorable location for large-volume excision to gain adequate surgical margins, especially for small tumors (34). Therefore, the low-risk group was safe enough and may gain limited benefit from RT.

In the present study, patients in the high-risk group were older age (>=65), had tumors in the inner or center location, and had larger tumor sizes, in whom RT conferred a survival benefit. Although elderly patients were more likely to have well-differentiated tumors, comorbid diseases and undertreatment associated with the domination of adherence to therapy regimens may lead to a worse prognosis (11, 32, 35). Similar to that in previous reports, our results showed that elderly patients (>=65) had a higher rate of non-BCSS (>=65 *vs*. <65, 66.9% *vs*. 54.7%) and a lower rate of acceptance of chemotherapy (>=65 *vs*. <65, 47.4% *vs*. 74.8%) among patients who did not receive RT after adjusting for confounders. In contrast to the low-risk group, for tumors in the inner quadrant or the center location, the limitation of excising a large amount of tissue, affects appearance, and more aggressive cancer characteristics may cause a higher risk of residual tumor cells (34, 36). Overall, we recommend routine RT after BCS for high-risk patients. Furthermore, for HER2+ elderly breast cancer patients included in the high-risk group, RT could improve significantly the OS and BCSS. Therefore, on the one hand, the management of comorbid diseases and treatment compliance should be strengthened to ensure tolerance and effectiveness of treatment (11). On the other hand, radiotherapy techniques with lower side effects should be sought on the premise of ensuring oncological outcomes. Current studies have shown that partial breast irradiation and targeted intraoperative radiotherapy might reduce the side effects of RT without affecting the survival of breast cancer patients compared with whole breast radiotherapy after BCS (37–39).

There was also a significant proportion of patients in the intermediate-risk group. RT could not prolong their BCSS, while the OS was improved. For this result, the possibility of a selection effect cannot be ruled out. Additionally, given the retrospective nature of this study, other unknown factors might have contributed to the results. Consequently, the decision to receive RT for these patients should not be made hastily, and follow-up prospective trials are urgently needed to assist clinical decision-making.

A better selection of patients at very low risk could be combined with the detection of radio-resistance of HER2+ subtype breast cancer cells. In the context of RT resistance, clinicians have more evidence to discuss the omission of RT for low-risk groups. And for HER2+ patients with high-risk characteristics, studies that were associated with whether the radiation boost or the transformation of RT technology could be reversed were still limited. A recent report based on dual blockade of CD47 and HER2 shows that the efficacy of RT can be enhanced by targeted therapy (21).

Our findings may provide a new perspective for the individualized treatment on HER2+ breast cancer patients after BCS. The study had several strengths: a large population, an assessment of non-BCSS based on Fine-Gray competing risk analysis, and PSM for adjusting confounders. More importantly, this is the first study, to our knowledge, to purely describe the population-level survival benefit from RT of HER2+ patients, and strict stratification analysis for nomogram construction with good internal validation was performed. Zhong Y et al. recently proposed that omitting RT for HER2+ patients older than 70 years who did not undergo axillary surgery is safe. Regrettably, they only focused on elderly patients, and no further estimation of the influence of non-BCSS (40).

Despite these promising results, limitations remain in our study. First, the absence of detailed information on anti-HER2 treatment, hormone therapy, and RT dose in the SEER database from 2010 to 2015. Although PSM was used to adjust for potential confounders, there were inevitably some unknown factors that still interfered with the study results. Nevertheless, we feel that, from monotherapy to dual anti-HER2 therapy and tyrosine kinase inhibitor (TKI), the number and quality of anti-HER2 therapies in slightly more than a decade have profoundly improved, which, as well as combinations with effective chemotherapy, significantly lowered the risk of LRR and effectively improved the survival of HER2+ early-stage patients (17). Therefore, the HER2+ population exempted from RT in this study is more likely to be the current and future candidate population not recommended for postoperative RT. A further limitation of our study was a relatively short median follow-up (31 months in low-risk group patients), although Kaplan-Meier analysis could be corrected by censored survival data. This may affect the survival estimates of early-stage young breast cancer patients. Therefore, long-term follow-up randomized controlled trials are needed for further verification.

In summary, we hope that our findings lay the foundation for future prospective clinical trials, which could identify more important prognostic factors and take improved contemporary systemic therapies into account to provide better-individualized recommendations for RT in HER2 patients after BCS.

## 5 Conclusion

RT could significantly improve the OS and BCSS of HER2+ early-stage breast cancer patients after BCS on the whole. For high-risk patients, RT is an essential component of cancer therapy. However, the omission of radiotherapy may be considered for low-risk HER2+ early-stage patients. Further validation and improvement of the nomogram by prospective study or randomized controlled trials are warranted.

## Data availability statement

Publicly available datasets were analyzed in this study. This data can be found here: http://seer.cancer./seerstat.

## Author contributions

DZ and WX contributed to the study’s conception and design. Material preparation, data collection, and analysis were performed by HY, MQ and YF. The first draft of the manuscript was written by HY and all authors commented on previous versions of the manuscript. All authors contributed to the article and approved the submitted version.

## References

[1] Boyages J. Radiation therapy and early breast cancer: current controversies. Med J Aust (2017) 207(5):216–22. doi: 10.5694/mja16.01020 28987136

[2] Clarke M Collins R Darby S Davies C Elphinstone P Evans V. Effects of radiotherapy and differences in the extent of surgery for early breast cancer on local recurrence and 15-year survival: An overview of the randomized trials. Lancet (2005) 366(9503):2087–106. doi: 10.1016/S0140-6736(05)67887-7 16360786

[3] Favourable and unfavorable effects on long-term survival of radiotherapy for early breast cancer: An overview of the randomized trials. early breast cancer trialists’ collaborative group. Lancet (2000) 355(9217):1757–70. 10832826

[4] Darby S McGale P Correa C Taylor C Arriagada R Clarke M. Effect of radiotherapy after breast-conserving surgery on 10-year recurrence and 15-year breast cancer death: A meta-analysis of individual patient data for 10,801 women in 17 randomised trials. Lancet (2011) 378(9804):1707–16. doi: 10.1016/S0140-6736(11)61629-2 PMC3254252 22019144

[5] Kunkler IH Williams LJ Jack WJL Cameron DA Dixon JM. Breast-conserving surgery with or without irradiation in women aged 65 years or older with early breast cancer (PRIME II): A randomised controlled trial. Lancet Oncol (2015) 16(3):266–73. doi: 10.1016/S1470-2045(14)71221-5 25637340

[6] Speers C Pierce LJ. Postoperative radiotherapy after breast-conserving surgery for early-stage breast cancer: A review. JAMA Oncol (2016) 2(8):1075–82. doi: 10.1001/jamaoncol.2015.5805 27243924

[7] Cui Y Li B Pollom EL Horst KC Li R. Integrating radiosensitivity and immune gene signatures for predicting benefit of radiotherapy in breast cancer. Clin Cancer Res (2018) 24(19):4754–62. doi: 10.1158/1078-0432.CCR-18-0825 PMC6168425 29921729

[8] Senkus-Konefka E Jassem J. Complications of breast-cancer radiotherapy. Clin Oncol (R Coll Radiol) (2006) 18(3):229–35. doi: 10.1016/j.clon.2005.11.004 16605054

[9] Citrin DE. Recent developments in radiotherapy. N Engl J Med (2017) 377(11):1065–75. doi: 10.1056/NEJMra1608986 28902591

[10] Hughes KS Schnaper LA Bellon JR Cirrincione CT Berry DA McCormick B. Lumpectomy plus tamoxifen with or without irradiation in women age 70 years or older with early breast cancer: Long-term follow-up of CALGB 9343. J Clin Oncol (2013) 31(19):2382–7. doi: 10.1200/JCO.2012.45.2615 PMC3691356 23690420

[11] Biganzoli L Battisti NML Wildiers H McCartney A Colloca G Kunkler IH. Updated recommendations regarding the management of older patients with breast cancer: A joint paper from the European society of breast cancer specialists (EUSOMA) and the international society of geriatric oncology (SIOG). Lancet Oncol (2021) 22(7):e327–e40. doi: 10.1016/S1470-2045(20)30741-5 34000244

[12] Gradishar WJ Anderson BO Balassanian R Blair SL Burstein HJ Cyr A. NCCN guidelines insights: Breast cancer, version 1.2017. J Natl Compr Canc Netw (2017) 15(4):433–51. doi: 10.6004/jnccn.2017.0044 28404755

[13] Gradishar WJ Moran MS Abraham J Aft R Agnese D Allison KH. Breast cancer, version 3.2022, NCCN clinical practice guidelines in oncology. J Natl Compr Canc Netw (2022) 20(6):691–722. doi: 10.6004/jnccn.2022.0030 35714673

[14] Killander F Karlsson P Anderson H Mattsson J Holmberg E Lundstedt D. No breast cancer subgroup can be spared postoperative radiotherapy after breast-conserving surgery. Fifteen-year results from the Swedish breast cancer group randomised trial, SweBCG 91 RT. Eur J Cancer (2016) 67:57–65. doi: 10.1016/j.ejca.2016.08.001 27614164

[15] Harbeck N Gnant M. Breast cancer. Lancet (2017) 389(10074):1134–50. doi: 10.1016/S0140-6736(16)31891-8 27865536

[16] Romond EH Perez EA Bryant J Suman VJ Geyer CE Davidson NE. Trastuzumab plus adjuvant chemotherapy for operable HER2-positive breast cancer. N Engl J Med (2005) 353(16):1673–84. doi: 10.1056/NEJMoa052122 16236738

[17] Loibl S Gianni L. HER2-positive breast cancer. Lancet (2017) 389(10087):2415–29. doi: 10.1016/S0140-6736(16)32417-5 27939064

[18] Sjöström M Lundstedt D Hartman L Holmberg E Killander F Kovács A. Response to radiotherapy after breast-conserving surgery in different breast cancer subtypes in the Swedish breast cancer group 91 radiotherapy randomized clinical trial. J Clin Oncol (2017) 35(28):3222–9. doi: 10.1200/JCO.2017.72.7263 28759347

[19] Formenti SC Spicer D Skinner K Cohen D Groshen S Bettini A. Low HER2/neu gene expression is associated with pathological response to concurrent paclitaxel and radiation therapy in locally advanced breast cancer. Int J Radiat Oncol Biol Phys (2002) 52(2):397–405. doi: 10.1016/S0360-3016(01)02655-4 11872285

[20] Kim H-A Kim E-K Kim M-S Yu J-H Lee M-R Lee HK. Association of human epidermal growth factor receptor 2 with radiotherapy resistance in patients with T1N0M0 breast cancer. J Breast Cancer (2013) 16(3):266–73. doi: 10.4048/jbc.2013.16.3.266 PMC3800722 24155755

[21] Candas-Green D Xie B Huang J Fan M Wang A Menaa C. Dual blockade of CD47 and HER2 eliminates radioresistant breast cancer cells. Nat Commun (2020) 11(1):4591. doi: 10.1038/s41467-020-18245-7 32929084 PMC7490264

[22] Edge SB Compton CC. The American joint committee on cancer: the 7th edition of the AJCC cancer staging manual and the future of TNM. Ann Surg Oncol (2010) 17(6):1471–4. doi: 10.1245/s10434-010-0985-4 20180029

[23] Benedetto U Head SJ Angelini GD Blackstone EH. Statistical primer: propensity score matching and its alternatives. Eur J Cardiothorac Surg (2018) 53(6):1112–7. doi: 10.1093/ejcts/ezy167 29684154

[24] Schuster NA Hoogendijk EO Kok AAL Twisk JWR Heymans MW. Ignoring competing events in the analysis of survival data may lead to biased results: a nonmathematical illustration of competing risk analysis. J Clin Epidemiol (2020) 122:42–8. doi: 10.1016/j.jclinepi.2020.03.004 32165133

[25] Denduluri N Somerfield MR Chavez-MacGregor M Comander AH Dayao Z Eisen A. Selection of optimal adjuvant chemotherapy and targeted therapy for early breast cancer: ASCO guideline update. J Clin Oncol (2021) 39(6):685–93. doi: 10.1200/JCO.20.02510 33079579

[26] Duru N Fan M Candas D Menaa C Liu H-C Nantajit D. HER2-associated radioresistance of breast cancer stem cells isolated from HER2-negative breast cancer cells. Clin Cancer Res (2012) 18(24):6634–47. doi: 10.1158/1078-0432.CCR-12-1436 PMC3593096 23091114

[27] Hou J Zhou Z Chen X Zhao R Yang Z Wei N. HER2 reduces breast cancer radiosensitivity by activating focal adhesion kinase *in vitro* and in vivo. Oncotarget (2016) 7(29):45186–98. doi: 10.18632/oncotarget.9870 PMC5216715 27286256

[28] Kim J-S Kim H-A Seong M-K Seol H Oh JS Kim E-K. STAT3-survivin signaling mediates a poor response to radiotherapy in HER2-positive breast cancers. Oncotarget (2016) 7(6):7055–65. doi: 10.18632/oncotarget.6855 PMC4872768 26755645

[29] Johnson ME Handorf EA Martin JM Hayes SB. Postmastectomy radiation therapy for T3N0: a SEER analysis. Cancer (2014) 120(22):3569–74. doi: 10.1002/cncr.28865 PMC4413466 24985911

[30] Kim HJ Han W Yi OV Shin HC Ahn S-K Koh BS. Young age is associated with ipsilateral breast tumor recurrence after breast conserving surgery and radiation therapy in patients with HER2-positive/ER-negative subtype. Breast Cancer Res Treat (2011) 130(2):499–505. doi: 10.1007/s10549-011-1736-3 21853352

[31] van de Water W Markopoulos C van de Velde CJH Seynaeve C Hasenburg A Rea D. Association between age at diagnosis and disease-specific mortality among postmenopausal women with hormone receptor-positive breast cancer. JAMA (2012) 307(6):590–7. doi: 10.1001/jama.2012.84 22318280

[32] Hsu CD Wang X Habif DV Ma CX Johnson KJ. Breast cancer stage variation and survival in association with insurance status and sociodemographic factors in US women 18 to 64 years old. Cancer (2017) 123(16):3125–31. doi: 10.1002/cncr.30722 28440864

[33] Yuan R Zhang C Li Q Ji M He N. The impact of marital status on stage at diagnosis and survival of female patients with breast and gynecologic cancers: A meta-analysis. Gynecol Oncol (2021) 162(3):778–87. doi: 10.1016/j.ygyno.2021.06.008 34140180

[34] Cantürk NZ Şimşek T Özkan Gürdal S. Oncoplastic breast-conserving surgery according to tumor location. Eur J Breast Health (2021) 17(3):220–33. doi: 10.4274/ejbh.galenos.2021.2021-1-2 PMC8246052 34263149

[35] Chesney TR Coburn N Mahar AL Davis LE Zuk V Zhao H. All-cause and cancer-specific death of older adults following surgery for cancer. JAMA Surg (2021) 156(7):e211425. doi: 10.1001/jamasurg.2021.1425 33978695 PMC8117065

[36] Kroman N Wohlfahrt J Mouridsen HT Melbye M. Influence of tumor location on breast cancer prognosis. Int J Cancer (2003) 105(4):542–5. doi: 10.1002/ijc.11116 12712447

[37] Vaidya JS Vaidya UJ Baum M Bulsara MK Joseph D Tobias JS. Global adoption of single-shot targeted intraoperative radiotherapy (TARGIT-IORT) for breast cancer-better for patients, better for healthcare systems. Front Oncol (2022) 12:786515. doi: 10.3389/fonc.2022.786515 36033486 PMC9406153

[38] Vaidya JS Bulsara M Baum M Wenz F Massarut S Pigorsch S. New clinical and biological insights from the international TARGIT-a randomised trial of targeted intraoperative radiotherapy during lumpectomy for breast cancer. Br J Cancer (2021) 125(3):380–9. doi: 10.1038/s41416-021-01440-8 PMC8329051 34035435

[39] Meattini I Marrazzo L Saieva C Desideri I Scotti V Simontacchi G. Accelerated partial-breast irradiation compared with whole-breast irradiation for early breast cancer: Long-term results of the randomized phase III APBI-IMRT-Florence trial. J Clin Oncol (2020) 38(35):4175–83. doi: 10.1200/JCO.20.00650 32840419

[40] Zhong Y Xu Y Zhou Y Mao F Lin Y Guan J. Breast-conserving surgery without axillary lymph node surgery or radiotherapy is safe for HER2-positive and triple-negative breast cancer patients over 70 years of age. Breast Cancer Res Treat (2020) 182(1):117–26. doi: 10.1007/s10549-020-05686-3 32430680

