# Supplementary material for: The role of radiotherapy in HER2+ early-stage breast cancer patients after breast-conserving surgery
Source: Front Oncol. 2023 Jan 4;12:903001. doi: 10.3389/fonc.2022.903001 (PMC9845557; doi:10.3389/fonc.2022.903001)
Supplement: Supplementary file 1 [file DataSheet_1.pdf]

Supplementary Material

The Role of Radiotherapy in HER2+ Early-stage Breast Cancer patients after Breast-Conserving Surgery

Huanzuo Yang[1†], MD, Mengxue Qiu[1†], MD, Yu Feng[1†], MD, Nan Wen [1], MD, Jiao Zhou[1], MD, Xiangquan Qin[1], MD, Juan Li[1], MD, Xinran Liu[1], MD, Xiaodong Wang[1,2]*, MD, Zhenggui Du[1,2]*, MD

[1] Breast Disease Center, West China Hospital, Sichuan University，37 Guoxue Street, Chengdu, 610041, China.

[2] Clinical Research Center for Breast, West China Hospital, Sichuan University， 37 Guoxue Street, Chengdu, 610041, China.

† These authors have contributed equally to this work and share the first authorship.

* Correspondence:

Zhenggui Du, MD, docduzg@163.com; Xiaodong Wang, MD, wxd65112@163.com.、

1. Supplementary Figures and Tables

1.1 Supplementary Figures

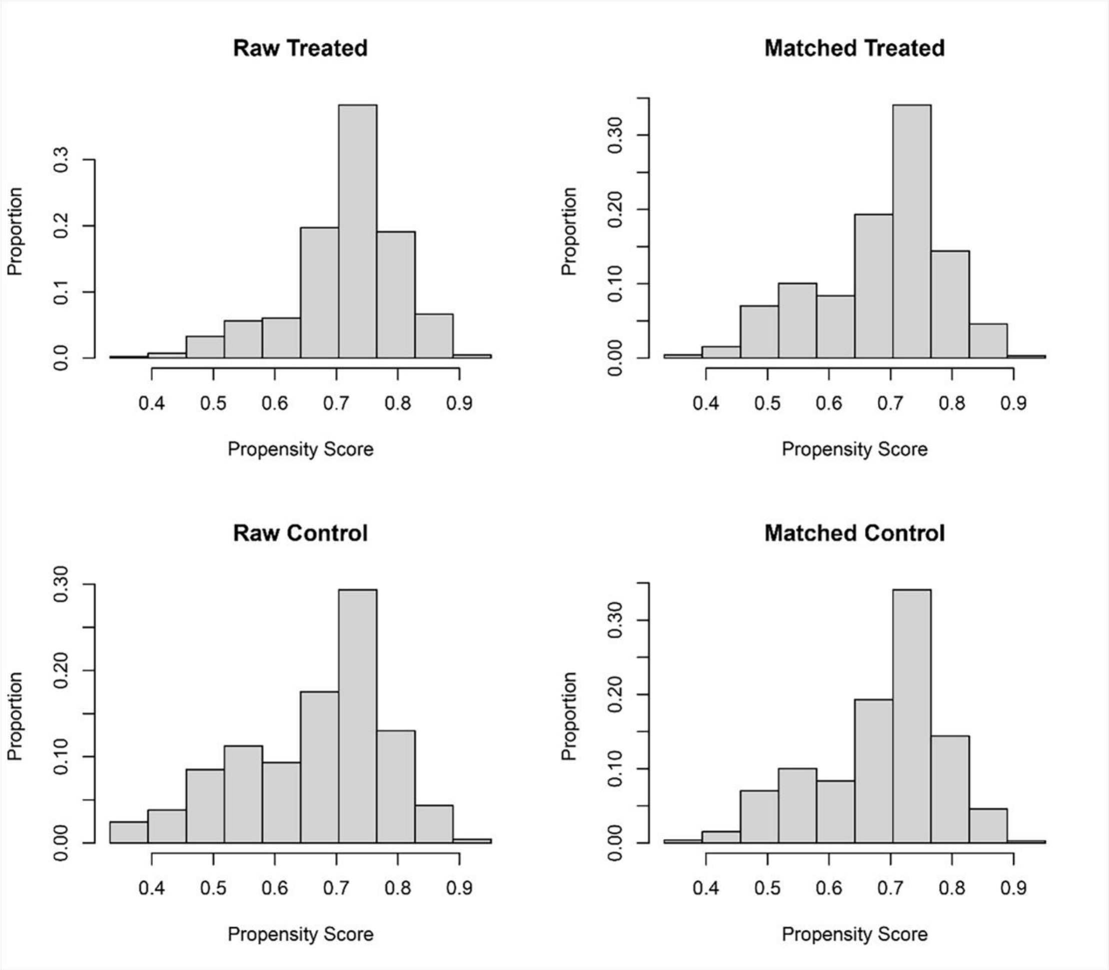

Supplemental Fig. 1 Histogram about distribution of propensity scores for matched and unmatched patients

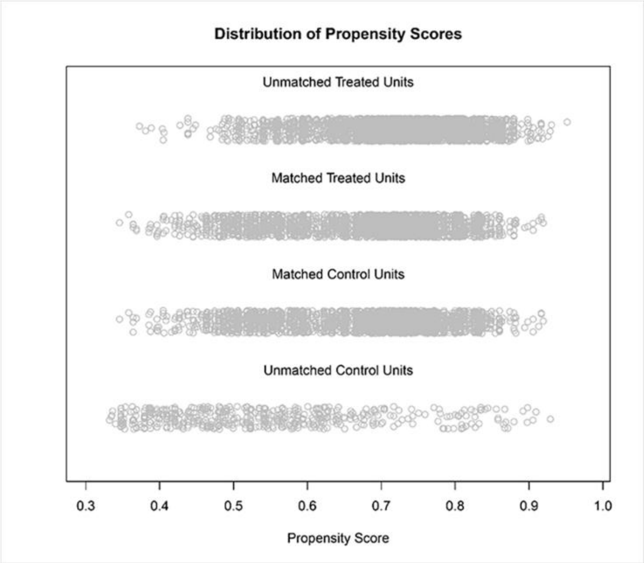

Supplemental Fig. 2 Jitter plot about distribution of propensity scores for matched and unmatched patients

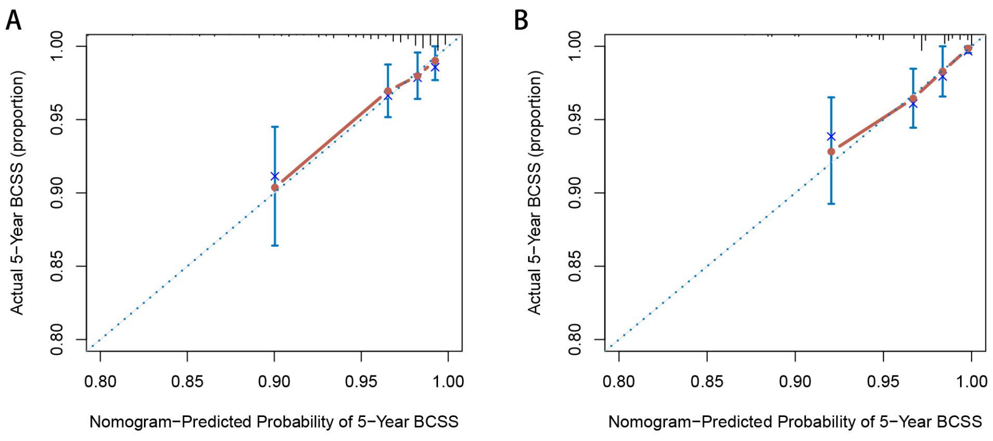

Supplemental Fig. 3 Calibration curves for 5-year BCSS in HER2+ patients with early-stage breast cancer after BCS[(A)internal validation of 5-year BCSS in no RT group;(B) external validation of 5-year BCSS in RT group]. The blue dotted line represents the ideal reference, which means that the nomogram-predicted survival probabilities (x-axis) exactly match the actual survival probabilities (y-axis). Red dots represent the nomogram-predicted probabilities for each group, and blue error bars represent the 95% CIs of these estimates.

1.2 Supplementary Tables

Supplemental Table 1 Demographic and disease characteristics of patients after PSM

Variables	All patients	no Radiotherapy	Radiotherapy	P-value*
	（n=5500）	(n=2750) n (%)	(n=2750) n (%)	
Age，years				0.796
<=40	204(3.7)	105(3.8)	99(3.6)	
40-65	3201（58.2）	1608(58.5)	1593(57.9)	
>=65	2095（38.1）	1037(37.7)	1058(38.5)	
Race				0.302
White	4243（77.1）	2148（78.1）	2095（76.2）	
Blake	572（10.4）	276（10.0）	296 (10.8)	
AIA	29（0.5）	16（0.6）	13（0.5）	
API	656（11.9）	310（11.3）	346 (12.6)	
Marital status				0.913
Unmarried	2247(40.9)	1126(40.9)	1121(40.8)	

Married	3253(59.1)	1624(59.1)	1629(59.2)	
Laterality				0.466
Right	2650(48.2)	1311(47.7)	1339(48.7)	
Left	2850(51.8)	1439(52.3)	1411（51.3）	
Tumor location				0.472
Outer quadrant	2380（43.3）	1216(44.2)	1164(42.3)	
Inner quadrant	1151（20.9）	571(20.8)	580(21.1)	
Center	186(3.4)	87(3.2)	99(3.6)	
Others [a]	1783(32.4)	876(31.9)	907(33.0)	
Tumor size, cm				0.789
<=0.5	493(9.0)	247(9.0)	246(8.9)	
0.5-1.0	1007(18.3)	489(17.8)	518(18.8)	
1.0-2.0	2403(43.7)	1212(44.1)	1191(43.3)	
2.0+	1597(29.0)	802(29.1)	795(28.9)	
Histology, ICD-O3				0.137
IDC	4870(88.5)	2456(89.3)	2414(87.8)	
ILC	148(2.7)	75(2.7)	73(2.7)	
IDC+ILC	137(2.5)	57(2.1)	80(2.9)	
Others [b]	345(6.3)	162(5.9)	183(6.7)	
Grade				0.117
Well, I	451(8.2)	210(7.6)	241(8.8)	
Moderately, II	2200(40.0)	1082(39.3)	1118(40.7)	
Poorly, III/IV	2849(51.8)	1458(53.0)	1391(50.6)	
Subtype				0.250
HER2+/HR+	4118(74.9)	2040(74.2)	2078(75.6)	
HER2+/HR-	1382(25.1)	710(25.8)	672(24.4)	
ER				0.200
Negative	1459(26.5)	751(27.3)	708(25.7)	
Positive	4041(73.5)	1999(72.7)	2042(74.3)	
PR				1.000
Negative	2402(43.7)	1201(43.7)	1201(43.7)	
Positive	3098(56.3)	1549(56.3)	1549(56.3)	
Stage, AJCC 7th				0.859
IA+0	3903(71.0)	1948(70.8)	1955(71.1)	

IIA	1597(29.0)	802(29.2)	795(28.9)	
T				0.958
Tis+T1mic	84(1.5)	41(1.5)	43(1.6)	
T1	3819(69.4)	1907(69.3)	1912(69.5)	
T2	1597(29.0)	802(29.2)	795(28.9)	
Chemotherapy				0.736
No/Unknown	1967(35.8)	977(35.5)	990(36.0)	
Yes	3533(64.2)	1773(64.5)	1760(64.0)	
Axillary Surgery				0.373
None	258(4.7)	140(5.1)	118(4.3)	
SLNB	4914(89.3)	2446(88.9)	2468(89.7)	
ALND	328(6.0)	164(6.0)	164(6.0)	

Abbreviations: RT, radiotherapy; AIA, American Indian/Alaska Native; API, Asian or Pacific Islander; ILC, invasive lobular carcinoma; HER2, human epidermal growth factor receptor 2; HR, Hormone receptor; −, negative; +, positive; ER, estrogen receptor; PR, progesterone receptor; IDC, invasive ductal carcinoma; SLNB, sentinel lymph node biopsy; ALND, axillary lymph node dissection.

a "others" includes "tumor location, NOS" and "overlapping lesion of breast such as 3, 6, 9, 12 o'clock" as recorded in the SEER database.

b "others" means histological types other than above four types.

Supplemental Table 2 Multivariate Cox model for HER2+ breast cancer patients after PSM

Variables	OS		BCSS	
	HR (95%CI)	P-value [b]	HR (95%CI)	P-value [b]
Age at diagnosis, y		<0.001		<0.001
<=40	1.00 [Reference]		1.00 [Reference]	
40-65	2.45(0.60-10.01)	0.21	1.14(0.271-4.764)	0.86
>=65	6.95(1.71-28.25)	0.007	2.94(0.70-12.31)	0.14
Race		0.01		0.001
White	1.00 [Reference]		1.00 [Reference]	
Black	1.21(0.81-1.79)	0.35	1.37(0.77-2.44)	0.29
AIA	2.68(0.85-8.42)	0.09	4.26(1.03-17.55)	0.05
API	0.46(0.24-0.87)	0.02	0.10(0.01-0.75)	0.03
Marital				
unmarried	1.00 [Reference]			
married	0.59(0.45-0.76)	<0.001		
Tumor Location				0.01
Outer quadrant			1.000 [Reference]	

Inner quadrant			1.69(1.06-2.69)	0.03
Center			1.07(0.38-3.01)	0.90
Others [a]			0.62()0.36-1.07)	0.09
Tumor size, cm		<0.001		<0.01
<=0.5	1.00 [Reference]		1.00 [Reference]	
0.5-1.0	1.80（0.95-3.44）	0.07	2.33(0.50-10.85)	0.28
1.0-2.0	2.20（1.20-4.04）	0.001	4.53(1.08-18.89)	0.04
2.0+	3.63（1.97-6.67）	0.001	9.08(2.18-37.86)	0.002
Subtype				
HER2+/HR+			1.00 [Reference]	
HER2+/HR-			1.79(1.17-2.74)	0.007
Chemotherapy				
No/Unknow	1.00 [Reference]		1.00 [Reference]	
Yes	0.53(0.40-0.70)	<0.001	0.55(0.36-0.84)	0.005
Radiotherapy				
No/Unknown	1.00 [Reference]		1.00 [Reference]	
Yes	0.45(0.35-0.58)	<0.001	0.53(0.35-0.80)	0.002
Axillary Surgery		<0.001		
None	1.00 [Reference]			
SLNB	0.35(0.25-0.50)	<0.001		
ALND	0.44(0.25-0.79)	0.006		

Abbreviations: AIA, American Indian/Alaska Native; API, Asian or Pacific Islander; ILC, invasive lobular carcinoma; HER2, human epidermal growth
factor receptor 2; HR, Hormone receptor; −, negative; +, positive; ER, estrogen receptor; PR, progesterone receptor; IDC, invasive ductal carcinoma; SLNB, sentinel lymph node biopsy; ALND, axillary lymph node dissection.

a “others” includes “tumor location, NOS” and “overlapping lesion of breast such as 3, 6, 9, 12 o’clock” as recorded in the SEER database.

b P < 0.05 was considered statistically significant.

Supplemental Table 3 outcome of the Fine-Gray test before and after PSM

Variables	Before PSM		After PSM	
	HR (95%CI)	P[a]	HR (95%CI)	P[a]
BCSS				
No RT	1.000 [Reference]		1.000 [Reference]	
RT	0.30 (0.22-0.42)	<0.001	0.53(0.35-0.80)	0.003
Non-BCSS				
No RT	1.000 [Reference]		1.000 [Reference]	
RT	0.27(0.21-0.35)	<0.001	0.43(0.31-0.60)	<0.001

Abbreviations: HR, hazard ratio; 95% CI, 95% confidence interval; PSM, propensity

score matching; BCSS, breast cancer-specific survival; non-BCSS, non-breast cancer specific survival
a P < 0.05 was considered statistically significant.

Supplemental Table 4 Cumulative incidence of BCSS and non-BCSS for total HER2+ breast cancer patients

Time of outcome, month	No Radiotherapy		Radiotherapy	
	Event of Interest: BCSS	Competing Event: non-BCSS	Event of Interest: BCSS	Competing Event: non BCSS
20	0.022	0.028	0.002	0.005
40	0.035	0.060	0.012	0.015
60	0.048	0.091	0.024	0.029

Abbreviations: BCSS, breast cancer-specific survival; non-BCSS, non-breast cancer-specific survival

Supplemental Table 5 Cumulative incidence of BCSS and non-BCSS for HER2+ breast cancer patients after PSM

Time of outcome, month	No Radiotherapy		Radiotherapy	
	Event of Interest: BCSS	Competing Event: non-BCSS	Event of Interest: BCSS	Competing Event: Non-BCSS
20	0.017	0.024	0.004	0.005
40	0.028	0.050	0.015	0.019
60	0.036	0.082	0.030	0.041

Abbreviations: BCSS, breast cancer-specific survival; non BCSS, non-breast cancer specific survival

Supplemental Table 6 BCSS-score of patients without RT after PSM

Variables	BCSS-score [b]
Age	
<=40	34
40-65	0
>=65	70
Marital	
Unmarried	40
Married	0
Tumor Location	
Outer quadrant	49
Inner quadrant	68

Center	100
Others[a]	0
Tumor size, cm	
<=0.5	0
0.5-1.0	36
1.0-2.0	58
2.0+	98
Chemotherapy	
No/Unknown	27
Yes	0

a "BCSS-score" means breast cancer-specific survival
b "Others" includes "tumor location, NOS" and "overlapping lesion of breast such as 3, 6, 9, 12 o'clock" as recorded in the SEER database.

Supplemental Table 7 C-Index for internal and external validation

Model	C-index (95% CI)
Internal validation	0.77 (0.71-083)
External validation	0.76 (0.69-0.83)

Abbreviations: 95% CI, 95% confidence interval; BCSS, breast cancer-specific survival
